# Supplementary material for: Regression-Based Normative Data for Independent and Cognitively Active Spanish Older Adults: Digit Span, Letters and Numbers, Trail Making Test and Symbol Digit Modalities Test
Source: Int J Environ Res Public Health. 2021 Sep 22;18(19):9958. doi: 10.3390/ijerph18199958 (PMC8507906; doi:10.3390/ijerph18199958)
Supplement: Supplementary file 1 [file ijerph-18-09958-s001.zip › ijerph-1339022-supplementary.pdf]

Supplementary material

Regression-Based Normative Data for Independent and Cognitively Active Spanish Older Adults: Digit Span, Letters and Numbers, Trail Making Test and Symbol Digit Modalities Test

Clara Iñesta[1], Javier Oltra-Cucarella[1,2,*], Beatriz Bonete-López[1,2], Eva Calderón-Rubio[1] and Esther Sitges-Maciá [1,]

Citation: Iñesta, C.; Oltra-Cucarella, J.; Bonete-López, B.; Calderón-Rubio, E.; Sitges-Maciá, E. Regression-based normative data for independent and cognitively active Spanish older adults: Digit Span, Letters and Numbers, Trail Making Test and Symbol Digit Modalities Test. *Int. J. Environ. Res. Public Health* **2021**, *18*, 9958. https://doi.org/10.3390/ijerph18199958

Academic Editor: Paul B. Tchounwou

Received: 29 July 2021
Accepted: 18 September 2021
Published: 22 September 2021

Publisher's Note: MDPI stays neutral with regard to jurisdictional claims in published maps and institutional affiliations.

Copyright: © 2021 by the authors. Licensee MDPI, Basel, Switzerland. This article is an open access article distributed under the terms and conditions of the Creative Commons Attribution (CC BY) license (http://creativecommons.org/licenses/by/4.0/).

Table S1. Comparing number of low scores between normative data sets (NEURONORMA-SABIEX)

		SABIEX		
		0	1+	Total
NEURONORMA	0	53	18	71
	1+	16	14	30
Total		69	32	101

Table S2. Comparing number of low scores between normative data sets (NEURONORMA-SABIEX) with TMT-B conditional on TMT-A

		SABIEX		
		0	1+	Total
NEURONORMA	0	55	16	71
	1+	16	14	30
Total		71	30	101

Table S3. Comparing number of low scores on TMT-B independent (TMT-B$_{NEURONORMA}$) and TMT-B conditional on TMT-A (TMT-B$_{SABIEX}$)

		TMT-B$_{SABIEX}$		
		SS7+	SS<7	Total
TMT-B$_{NEURONORMA}$	SS7+	84	5	89
	SS<7	13	0	13
Total		97	5	102
